# Myeloid-Derived Suppressor Cells in Kidney Transplant Recipients and the Effect of Maintenance Immunotherapy

**DOI:** 10.3389/fimmu.2020.00643

**Published:** 2020-04-30

**Authors:** María Iglesias-Escudero, David Sansegundo-Arribas, Paloma Riquelme, David Merino-Fernández, Sandra Guiral-Foz, Carmen Pérez, Rosalia Valero, Juan Carlos Ruiz, Emilio Rodrigo, Patricia Lamadrid-Perojo, James A. Hutchinson, Jordi Ochando, Marcos López-Hoyos

**Affiliations:** ^1^Transplantation and Autoimmunity Group, Marqués de Valdecilla Health Research Institute (IDIVAL) Santander, Spain; ^2^Department of Immunology, University Hospital Marqués de Valdecilla, Santander, Spain; ^3^Section of Experimental Surgery, Department of Surgery, University Hospital of Regensburg, Regensburg, Germany; ^4^Department of Nephrology, University Hospital Marqués de Valdecilla, Santander, Spain; ^5^Department of Oncological Sciences, Icahn School of Medicine at Mount Sinai, New York, NY, United States; ^6^Immunología de Trasplantes, Centro Nacional de Microbiología, Instituto de Salud Carlos III, Madrid, Spain

**Keywords:** kidney transplantation, mTOR inhibition, myeloid-derived suppressor cells, tacrolimus, immunosuppression

## Abstract

Myeloid-derived suppressor cells (MDSC) represent a heterogeneous group of myeloid regulatory cells that were originally described in cancer. Several studies in animal models point to MDSC as important players in the induction of allograft tolerance due to their immune modulatory function. Most of the published studies have been performed in animal models, and the data addressing MDSCs in human organ transplantation are scarce. We evaluated the phenotype and function of different MDSCs subsets in 38 kidney transplant recipients (KTRs) at different time points. Our data indicate that monocytic MDSCs (Mo-MDSC) increase in KTR at 6 and 12 months posttransplantation. On the contrary, the percentages of polymorphonuclear MDSC (PMN-MDSC) and early-stage MDSC (e-MDSC) are not significantly increased. We evaluated the immunosuppressive activity of Mo-MDSC in KTR and confirmed their ability to increase regulatory T cells (Treg) *in vitro*. Interestingly, when we compared the ability of Mo-MDSC to suppress T cell proliferation, we observed that tacrolimus, but not rapamycin-treated KTR, was able to inhibit CD4^+^ T cell proliferation *in vitro*. This indicates that, although mTOR inhibitors are widely regarded as supportive of regulatory responses, rapamycin may impair Mo-MDSC function, and suggests that the choice of immunosuppressive therapy may determine the tolerogenic pathway and participating immune cells that promote organ transplant acceptance in KTR.

## Introduction

Kidney transplantation is a treatment option for patients with end-stage renal disease (ESRD). Although immunosuppressive protocols have clearly reduced the incidence of acute rejection, transplant patients continue at high risk of treatment side effects, and long-term allograft survival has not improved significantly (1). As a consequence, the main goals in transplantation are to predict the risk of developing rejection and to find biomarkers of tolerance to allow immunosuppression withdrawal in order to minimize the adverse effects of the currently available immunosuppressive regimens.

An increasing field of research is focused on the study of immune cells with regulatory and/or suppressive function. Among them, myeloid-derived suppressor cells (MDSCs) have gained attention in the last years. The MDSCs are a heterogeneous group of myeloid cells able to suppress adaptive and innate immune responses and have been suggested as potential biomarkers for allograft tolerance. They were initially described in cancer, and several studies have pointed out MDSC to play an important role in the regulation of immune responses in other clinical setting, such as organ transplantation, infection, and autoimmune diseases (2–4).

Myeloid-derived suppressor cells were first described in mice as CD11b^+^ Gr1^+^ cells, and experimental transplant models demonstrated that MDSCs have an important role in the induction of tolerance (5). On the contrary, evidence on their role in human transplantation is scarce and non-conclusive. In renal transplant patients, Luan et al. observed MDSC, defined as CD33^+^ CD11b^+^HLA-DR^–^, able to expand T regulatory cells (Treg) *in vitro* and correlate with Treg cell numbers *in vivo* (6). These data were confirmed by Meng et al. who associated MDSC numbers with less tissue injury and longer allograft survival (7). Human MDSCs are divided into three main subsets: monocytic MDSC (Mo-MDSCs: CD33^+^CD11b^+^CD14^+^HLA-DR^–^), polymorphonuclear MDSC (PMN-MDSCs: CD33^+^CD11b^+^CD15^+^HLA-DR^–^), and a population lacking both differentiation surface markers classified as early-stage MDSC (e-MDSCs: CD33^+^HLA-DR^–^CD15^–^ CD14^–^) (8). Since these phenotypic markers are not exclusive of MDSCs and they are present in other myeloid cells such as monocytes, macrophages, and granulocytes, MDSC cells are further defined upon demonstration of their suppressive function (9).

Due to the paucity of the MDSC data in clinical organ transplantation and that different immunosuppressants may have a distinct effect on MDSC, we monitored circulating MDSC subset frequencies in kidney transplant recipients (KTRs). The main goal of the study was to compare transplant recipients receiving standard triple therapy to those maintained on a regimen including rapamycin and evaluate the effect of each therapeutic arm on MDSC in relation to kidney transplant outcomes.

## Materials and Methods

### Study Design

A total of 38 consecutive KTRs were enrolled in the study after giving consent while they were listed for kidney transplantation in the Hospital Universitario Marqués de Valdecilla in 2016. The study was approved by the Hospital Universitario Marqués de Valdecilla Ethics Committee. The mean follow-up time was 459 days. The clinical and immunological features of the KTR are summarized in Table 1. Clinical data were collected from patient records, and blood was drawn at baseline/day 0, 180, and 360 days after transplantation. The clinical and immunological features of the KTR are summarized in Table 1.

**TABLE 1:** Main features of study population (*N* = 38).

Recipients: Age, mean, years	51.88(SD13.23)
Donors: Age, mean, years	49.61(SD12.63)
Healthy controls: Age, mean, years	46.17(SD11.85)
Recipient Sex (% female)	18(47.37%)
Donor sex (% female)	19(50%)
Dialysis post kidney transplant	10(26%)
Preexisting anti-HLA antibodies	13(34.21%)
Class I antibodies	10(26%)
Class II antibodies	8(21.05%)
Rejection	6(15.78%)
RT	11(28.94%)
**Induction treatment**	
None	21(55.26%)
ATG	12(31.57%)
Basiliximab	5(13.15%)
Both	0(0.00%)
**Immunosupressive protocol**	
Calcineurin inhibitor	33(86.84%)
mTOR inhibitor	0(0.00%)
Both	5(13.15%)
**ABDR mismatches**	
>3	24(63.15%)
=3	14(36.84%)
**Class II mismatches**	
0	8(21.05%)
1	17(44.73%)
2	13(34.2%)
**Renal disease**	
Glomerular	11(28.94%)
Others	1(2.63%)
Congenital	7(18.42%)
Sistemic	10(26.31%)
Vascular	2(5.26%)
Interstitial	5(13.15%)
Unknown	2(5.26%)
**Peripheral blood creatinine**	
Cr 7 days post trasplant	2.28(SD1.70)
Cr 30 days post transplant	1.90(SD1.39)
Cr 120 days post transplant	1.40(SD0.45)
Cr 180 days post transplant	1.40(SD0.48)

*SD, standard deviation; ESRD, end stage renal disease; 1stT, first transplant; RT, retransplant patients.*

### Monoclonal Antibodies and Flow Cytometry Analysis

The PBMCs or isolated MDSCs were stained with the following monoclonal antibodies: anti-CD33-APC (clone D3HL60.251), anti-CD3-FITC (clone UCHT1), anti-CD14-ECD (clone RMO52), and anti-CD11b-PE-cyanin (clone Bear1) (Beckman Coulter, Marseille, France); anti-CD16-APC-Cy7 (clone 3G8) and anti-CD56-FITC (clone HCD56 and anti-HLA-DR-Brilliant Violet 510 (clone L243) (Biolegend, San Diego, CA, United States); anti-CD19-FITC (clone 4G7), anti-CD14-FITC (clone MφP9), anti-CD25-PE (clone 2A3), and anti-FoxP3-Pacific Blue (clone 206D) (BD Biosciences); anti-CD15-CF Blue (clone MCS-1) (Inmunostep, Salamanca, Spain); and anti-CD4-APC-Vio770 (clone REA623) from Miltenyi Biotech. The cells were incubated for 20 min, washed with phosphate-buffered saline (PBS), and acquired in a Cytoflex^®^ flow cytometer (Beckman Coulter). MDSCs were quantified by flow cytometry following the gating strategy proposed by Bronte et al. (8) to characterize MDSC subsets: Mo-MDSCs (CD33^+^CD11b^+^HLADR^–^ CD14^+^ CD15^–^), PMN-MDSC (CD33^+^CD11b^+^HLADR^–^ CD15^+^ CD14^–^), and e-MDSC Lin^–^ (CD14^+^CD56^+^CD3^+^CD19^+^) CD33^+^CD11b^+^HLADR^–^ CD14^–^CD15^–^. Total MDSCs were defined as CD33^+^CD11b^+^HLADR^–^ cells. Fluorescence minus one control was used to identify HLA-DR^+^ and HLA-DR^–^ cells.

### Isolation and Sorting of MDSC

Human peripheral blood mononuclear cells (PBMCs) were isolated from buffy coats from healthy donors and from KTR by Ficoll density gradient centrifugation. To isolate CD33^+^ HLA-DR^–^ and CD33^+^ HLA-DR^–^ CD14^+^ cells (Mo-MDSC), the CD33^+^ cells were first sorted by magnetic-automated cell sorting using CD33-positive separation microbeads (Miltenyi Biotech, Bergisch Gladbach, Germany) according to the manufacturer’s instructions. Further isolation of CD33^+^HLA-DR^–^ cells and CD33^+^HLA-DR^–^ CD14^+^ was performed by sorting enriched cells on a FACS-ARIA II (BD Biosciences, San Jose, CA, United States). The purity of the cell sorting was tested after each experiment, and >98% efficiency was considered acceptable for the study. The experimental conditions were replicated at least four times.

### Whole Blood Cultures

Whole blood culture was performed as follows: fresh blood anticoagulated with lithium-heparin was diluted 1:4 in Gibco^TM^ DMEMF/12 GlutaMAX^TM^ supplement medium (Thermo Fisher Scientific) containing 100 U/ml penicillin (Lonza) and 100 mg/ml streptomycin (Lonza). Cells were stimulated throughout the cultures with 5 ng/ml recombinant human monocyte colony stimulating factor (rhM-CSF; R&D Systems, Wiesbaden-Nordenstadt). For some experiments, human CD14^+^ monocytes were isolated from Ficoll density gradient centrifugation of PBMC followed by positive selection using anti-CD14 microbeads (Miltenyi, Bergisch-Gladbach, Germany). Isolated CD14^+^ monocytes were stained with Cell Tracker^TM^ Green CMFDA Dye (Thermo Fisher Scientific) at 2 nM and then added back into whole blood cultures at 10^5^ cells/tube (Falcon^®^ 5 ml round bottom polystyrene test tube) diluted 1/4 in Gibco^TM^ DMEMF/12 GlutaMAX^TM^ supplement medium (Thermo Fisher Scientific) and supplemented with 100 U/ml penicillin (Lonza), 100 mg/ml streptomycin (Lonza), and rhM-CSF (R&D Systems, Wiesbaden-Nordenstadt) at 5 ng/ml carried on 0.1% human albumin. Purity of sorted cells was tested after isolation, and >95% efficiency was considered acceptable for the study. Cells were collected, and location was analyzed at baseline and 1 and 2 days after culture.

### *In vitro* Evaluation of MDSC Suppressor Function

CD4^+^ T cells were isolated from healthy donors or KTR PBMC by immunomagnetic depletion using EasySep^TM^ Human CD4^+^ naive T Cell Isolation Kit (Stemcell Technologies, Grenoble, France) and incubated with carboxyfluorescein succinimidyl ester (CFSE). The CFSE-labeled T CD4^+^ cells (5 × 10^5^) were stimulated with Dynabeads human T-activator CD3/CD28 (Life Technologies AS, Oslo, Norway) in U-bottomed 96-well plates with complete Roswell Park Memorial Institute (RPMI) media supplemented with 10% human AB + serum. Proliferation was determined using flow cytometry. Autologous Mo-MDSCs were added to the culture at 1:2 ratio (CD4^+^ T cells: MDSCs), and proliferation was determined at day 5. Proliferation assays from blood donors were performed five times. These same functional assays were also carried out with MDSC from four renal transplant receptors: four patients under calcineurin inhibitor (tacrolimus) and four patients under mTOR inhibitor treatment (rapamycin) with at least 24 months of IS treatment.

### *In vitro* Expansion of Treg Generation

peripheral blood mononuclear cells were obtained from KTR under maintenance immunosuppression with tacrolimus. CD4^+^ T cells were sorted from the PBMC as described above. CD4^+^ T cells (5 × 10^5^) were polyclonally stimulated and cultured with CD33^+^HLA-DR^–^CD14^+^ (Mo-MDSC) at different concentrations. Treg generation was determined at day 5 by staining with the monoclonal antibodies indicated above and flow cytometry analysis.

### Western Blot

Gel electrophoresis and immunoblotting were performed as described elsewhere (10).

### Statistical Analysis

Non-parametric Mann–Whitney *U* test and Student’s *t*-test were used to compare two groups, as appropriate. More than two groups were compared using the parametric analysis of variance (ANOVA), the non-parametric Kruskal–Wallis (not matching), or Friedman (repeated measures) test. Comparisons between two paired groups were performed using the Student’s *t*-test for paired data or the Wilcoxon signed-rank test when data were or not normally distributed, respectively. Multiple comparisons were assessed using Dunn or Tukey’s tests. Statistical analyses were performed using GraphPad software version 6.01 (GraphPad Inc., San Diego, CA, United States). To examine the relationship between bivariate variables, the Pearson correlation was calculated using SPSS Statistics version 24.

## Results

### Monitoring MDSC in Kidney Transplant Patients

**FIGURE 1:**
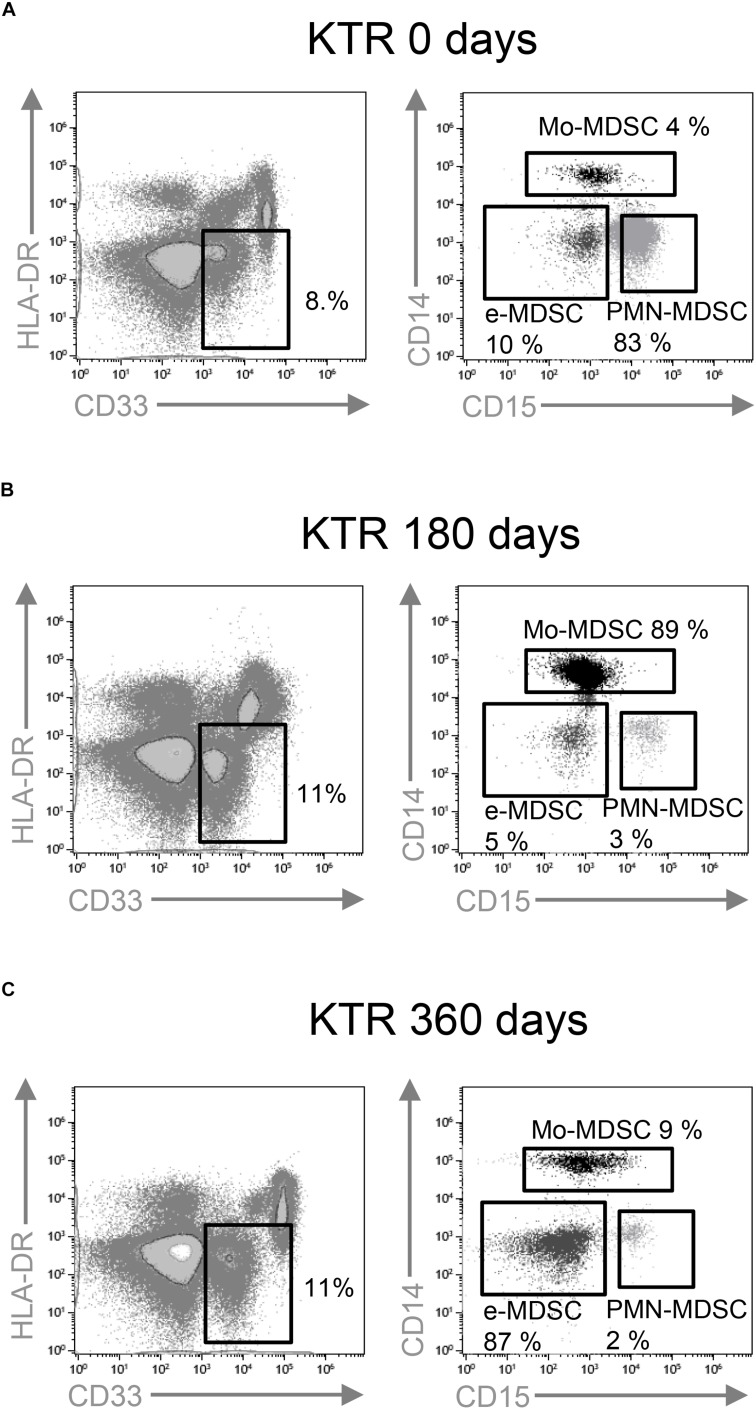
Characterization of myeloid-derived suppressor cell (MDSC) subsets by flow cytometry. CD33^+^ HLA-DR^–^ myeloid cells were selected from live cells after doublets and debris exclusion. To define monocytic (Mo-MDSC), early-stage (e-MDSC), and polymorphonuclear (PMN-MDSC) MDSC, the CD14 and CD15 expression was analyzed on cells selected from CD33^+^HLA-DR^–^ MDSC. Representative flow cytometry data of MDSC from **(A)** patients on the day of transplantation (day 0), **(B)** kidney transplant recipients on day 180, and **(C)** day 360 posttransplantation is shown.

**FIGURE 2:**
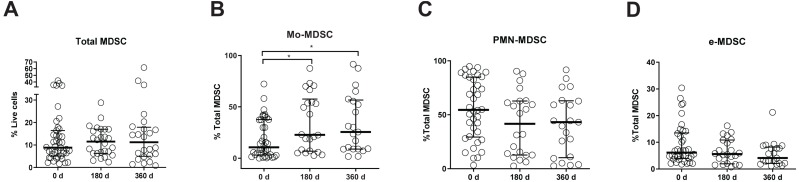
Myeloid-derived suppressor cell (MDSC) frequencies in kidney transplant recipients (KTRs). **(A)** Frequencies of total myeloid-derived suppressor cells (MDSC) in peripheral blood mononuclear cells (PBMC); **(B)** monocytic MDSC (Mo-MDSC); **(C)** early-stage MDSC (eMDSC); and **(D)** polymorphonuclear MDSC (PMN-MDSC) are shown. Differences between groups were assessed by Kruskal-Wallis and Mann-Whitney *U* test. (*p < 0.05).

We hypothesized that MDSC subset frequencies might serve as useful biomarkers of clinical outcome after kidney transplantation. Therefore, we first quantified Mo-MDSC, PMN-MDSCs, and e-MDSC in peripheral blood from KTRs at 0, 180, and 360 days after transplantation. We found an increase in total CD33^+^HDL-DR^lo^ MDSC frequency at 180 days after transplantation [median, 11.5%; interquartile range (IQR), 6.2–17.0%] (Figures 1B, 2A) in comparison with patients on the day of transplantation (median, 8.8%; IQR, 5.0–16.4%) (Figures 1A, 2A). MDSC frequency at 360 days posttransplant was also increased but not significantly (median, 11.2%; IQR, 4.9–17.8%; Figures 1C, 2A). Next, we examined changes in MDSC subset distribution after transplantation (Figure 2 and Supplementary Figures S1, S2). Mo-MDSC frequencies were significantly increased at 180 and 360 days posttransplant (median, 22.71%; IQR, 6.75–57.56% and median, 25.48%; IQR, 8.85–56.58%) in comparison to patients on the day of transplantation (median, 10.56%; IQR, 3.18–37.55%) (Figures 1A–C, 2B). PMN-MDSC and e-MDSC frequencies were lower at 180 days after transplantation (median, 41.71%; IQR, 12.67–62.79% and median, 5.5%; IQR, 1.9–10.87%) compared to patients on the day of transplantation (median, 54.6%; IQR, 29.4–84.95% and median, 6.15%; IQR, 3.9–13.5%), and they remained lower 360 days posttransplantation (median, 43.14%; IQR, 10.28–63.02% and median, 4.09%; IQR, 2.11–8.2%) (Figures 1A–C, 2C,D). Despite these changes, we did not find any association between the MDSC subsets, and the clinical data are summarized in Table 1 for patients included in the present work. Importantly, all the KTRs were receiving tacrolimus (Table 1) as main immunosuppressant during the first 360 days after transplantation shown.

### MDSC From Transplant Patients Induce the Production of Tregs *in vitro*

**FIGURE 3:**
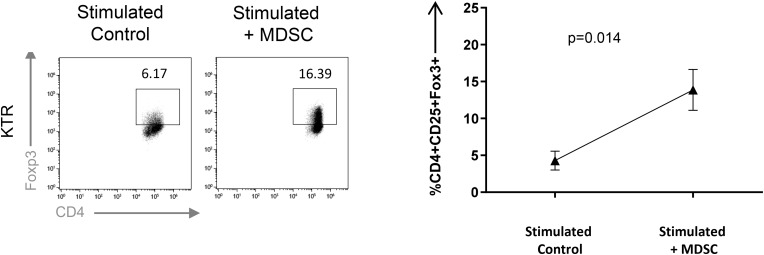
Monocytic myeloid-derived suppressor cell (Mo-MDSC) from kidney transplant recipients (KTRs) expand Treg *in vitro*. MDSC dependent CD4^+^FoxP3^+^ Treg expansion was analyzed by flow cytometry. Naive CD4^+^ T cells cocultured under polyclonal activation with autologous Mo-MDSC obtained KTR at day 360 are shown (*n* = 3, unpaired *t*-test).

Treg expansion is one of the main mechanisms by which MDSCs exert suppressive function (11, 12). Hence, we evaluated the capacity of Mo-MDSC from healthy donors and KTR to boost Tregs *in vitro.* We observed a significant increase in the frequency of Tregs recovered from the culture when CD4^+^ T cells were stimulated with Mo-MDSC from cells from KTR at 360 days after transplantation, confirming their suppressive function (Figure 3).

### MDSC From Tacrolimus Treated KTR Effectively Suppress T Cell Proliferation *in vitro*

**FIGURE 4:**
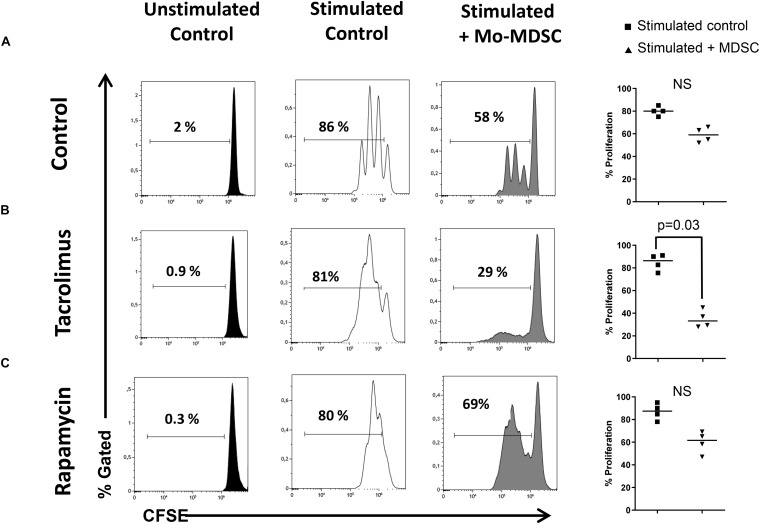
Suppressive function of myeloid-derived suppressor cells (MDSCs). Sorted CD4^+^ T cells were stained with carboxyfluorescein succinimidyl ester (CFSE) and cultured under polyclonal activation alone or with autologous monocytic myeloid-derived suppressor cells (Mo-MDSCs). Representative flow cytometry plots of four independent experiments with Mo-MDSCs from healthy volunteers; kidney transplant patients under tacrolimus treatment and rapamycin are shown. Individual data of experiments are displayed in the right plot graphs where stimulated control cells are represented as black squares and stimulated cells + Mo-MDSC are represented as black triangles. Differences between groups were assessed by Mann–Whitney test and only indicated if differences were significant.

The T-cell-suppressive capacity of Mo-MDSC from healthy controls, tacrolimus, and rapamycin-treated KTR was compared using an *in vitro* assay of polyclonally activated T cell proliferation. Sorted Mo-MDSC were added at a 1:2 ratio to autologous CD3/CD28-stimulated CD4^+^ T cells. Four patients under long-term tacrolimus treatment and four patients under long-term rapamycin maintenance therapy were analyzed (Figure 4). Results indicate that Mo-MDSC obtained from tacrolimus treated KTR were significantly suppressive in comparison with rapamycin treated KTR. This suggests that Mo-MDSC from transplant patients exhibit different suppressive function *in vitro*, according to the immunosuppressive therapy that KTRs receive.

### Rapamycin Inhibits the Function of *in vitro* Generated Myeloid Suppressor Cells

**FIGURE 5:**
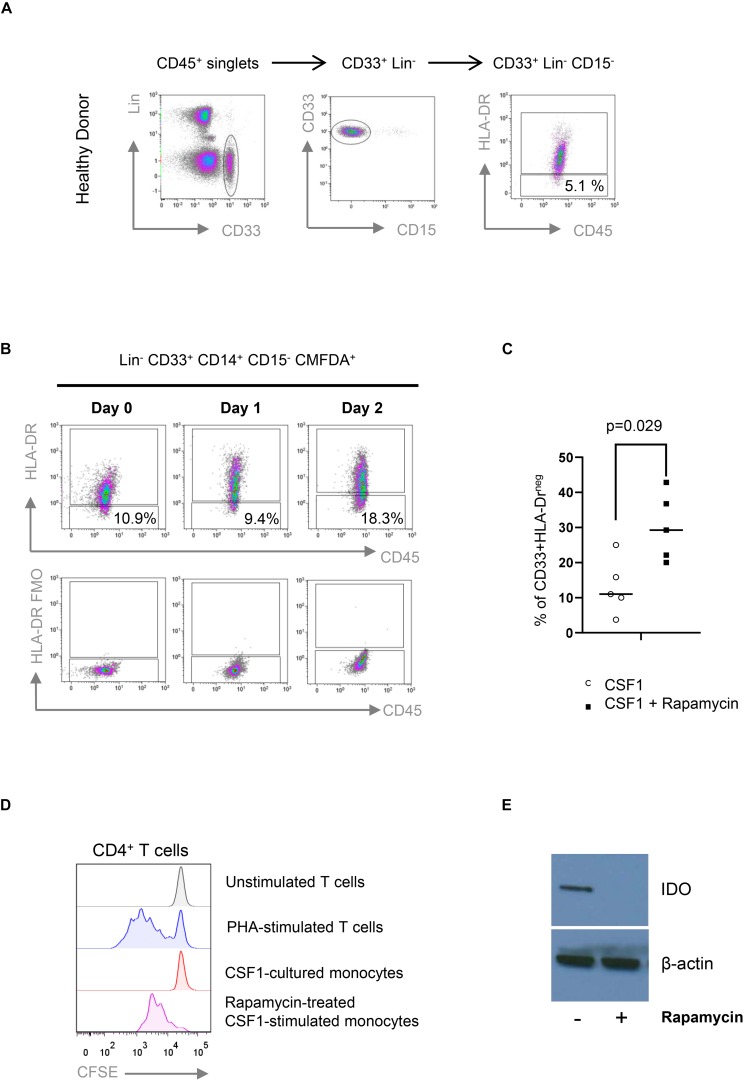
Rapamycin prevents the suppressive function of CD33^+^HLA-DR^–^/low myeloid cells. **(A)** Gating strategy for the identification of CD33^+^HLA-DR^–^/low myeloid cells obtained from healthy control (HC). Fluorescence minus one (FMO) controls were used to define HLA-DR expression (not shown). **(B)** Colony stimulating factor 1 (CSF1) induces the accumulation of CD33^+^HLA-DR^–^/low myeloid cells *in vitro*. CD14^+^ cells were isolated from peripheral blood, labeled with CFDMA and cocultured with CSF1 for 2 days. CD33^+^HLA-DR^–^/low phenotype was analyzed in CFDMA^+^ cells at day 0, 1, and 2 after culture. FMO controls were used to define HLA-DR expression. **(C)** CD33^+^HLA-DR^–^/low myeloid cell frequencies after 48 h in WB cultures treated with or without rapamycin. Differences between groups were assessed by paired *t*-test. **(D)** Rapamycin-treated CSF1-stimulated monocytes are less effective than untreated monocytes in suppressing phytohemagglutinin (PHA)-stimulated proliferation of allogeneic human CD4^+^ T cells in 1:1 direct cocultures (*n* = 3). **(E)** Western Blot analyses indicate that rapamycin-treated CSF1-derived CD33^+^HLA-DR^–^/low myeloid cells prevents the expression of IDO.

Following-up our observation of Mo-MDSC obtained from rapamycin-treated KTRs, we next investigated the effect of rapamycin on myeloid suppressor cells that were generated *in vitro* from whole blood cultures. First, we developed a flow cytometry panel that allowed us to reliably detect Mo-MDSC from human whole blood cultures according to their CD45^+^ CD33^+^ Lin^–^ HLA-DR^lo^ CD14^+^ CD15^–^ phenotype (Figure 5A). Using whole blood cultures, we next investigated whether CSF1-stimulated human monocytes acquire a Mo-MDSC phenotype (CD33^+^ Lin^–^ HLA-DR^lo^ CD14^+^ CD15^–^) *in vitro*. When cultured for 48 h, we observed an increase in Mo-MDSC frequency in whole blood cultures from healthy donors (Figure 5B). Next, we investigated the effect of rapamycin on Mo-MDSC in whole blood cultures and observed that rapamycin led to accumulation of HLA-DR^lo^ CD14^+^ Mo-MDSC over 48 h (Figure 5C). This suggests that mTOR inhibition promotes Mo-MDSC development. Surprisingly, we found that rapamycin exposure substantially reduced the T-cell-suppressive capacity of Mo-MDSC (Figure 5D). It has been previously shown that T cell suppression by human-monocyte-derived Mo-MDSC is in part mediated by the expression of the immunosuppressive molecule indoleamine 2,3-dioxygenase (IDO) (13). Our results confirm that rapamycin blocked the expression of IDO (Figure 5E), suggesting that the suppressive effect of Mo-MDSC from rapamycin-treated KTR may be compromised due to the impaired expression of IDO.

## Discussion

Myeloid-derived suppressor cells represent a varied group of myeloid regulatory cells that were originally studied in cancer (14). Several studies demonstrating their immunoregulatory action in animal models point to a potential role of MDSC in the induction of tolerance after transplantation (2). As most of the published studies were performed in animal models, there is a paucity of data addressing MDSC features and their role in human transplantation. We found that absolute numbers of circulating total MDSC were increased in KTR and in the short term after transplantation, whereas they declined to baseline levels 1 year after transplantation. We also observed an increase in Mo-MDSCs frequencies in the short term after transplantation and 1 year after transplantation. Luan et al. found that peripheral blood MDSCs were increased in KTR (6). Hock et al. also reported that renal transplant recipients had elevated frequencies of circulating MDSC (15), but they further found that MDSC numbers had returned to normal levels 12 months posttransplantation (16). However, in their previous study, long-term KTR had increased MDSC numbers, suggesting that MDSC recover and even expand in the long term, as graft acceptance progresses. These observational studies suggest that MDSC numbers increased rapidly and peaked following immunosuppressive therapy. Whether these increases are the result of potential differences between the two immunosuppressive regimens used (tacrolimus and mTOR inhibitors) or whether MDSC subsets are differentially regulated by local conditions or treatments is still a matter of debate.

Studies developed in mice suggest that MDSCs have an important role to induce T regulatory cells (Treg) after transplant (11, 12), but their role in human transplantation is still unclear. In KTR, Luan et al. observed that CD33^+^ CD11b^+^ HLA-DR^–^ MDSC are capable of expanding Treg, and they correlate with Treg increases *in vivo* (6). Consistent with this view, Meng et al. (7) found that MDSCs isolated from transplant recipients were also able to expand regulatory T cells and were associated with longer allograft survival. Okano S. et al. also found a positive correlation between MDSC and Treg in intestinal transplant patients (17), and we report here an increase in Treg expansion after Mo-MDSC coculture. However, there was no significant linear association between MDSC absolute numbers and percentage Treg when we examined the relationship between total MDSC subsets and CD4^+^CD25^+^Foxp3^+^ Treg *in vivo*.

Myeloid cell surface markers define potential MDSC, but the lack of unique phenotypic markers obliges to perform functional studies to identify MDSC subsets. We tested the suppressive capacity of MDSCs from KTR under calcineurin (tacrolimus) or mTOR (rapamycin) inhibition at 360 days of immunosuppressant maintenance therapies. Our results demonstrate that MDSC from healthy donors display marginal suppression of polyclonal T CD4^+^ responses. In contrast, Mo-MDSCs from KTR exhibit potent suppressive function. The results are consistent with previous data demonstrating that CD11b^+^CD33^+^HLA-DR^–^ myeloid cells from human KTR inhibit T cell proliferation, but they found no inhibition when CD11b^+^CD33^+^HLA-DR^–^ cells were obtained from healthy donors (6). Moreover, we observed that Mo-MDSC from KTR under tacrolimus treatment had increased suppressive activity compared to rapamycin, and this immune inhibitory function may be related to the upregulation of inducible nitric oxide synthase (iNOS) (18).

On the other hand, rapamycin downregulates iNOS expression in MDSC, and the suppressive activity and MDSC numbers are significantly reduced after rapamycin treatment in an allogeneic skin transplant model (19). Our results are consistent with this hypothesis, and we attribute loss of suppressive function to diminished IDO expression in rapamycin-exposed Mo-MDSC. However, other studies demonstrated that rapamycin prolongs cardiac allograft survival through the enhancement of MDSC migration and suppressive activity (20). Chen X. et al. showed that mTOR signaling is a negative determinant of MDSC function in immune-mediated hepatic injury (IMH) diseases. In the context of IMH, the blocking of mTOR with rapamycin or mTOR-deficient CD11b^+^Gr1^+^ MDSC mediated the protection against IMH (21). Another study addressing the murine MDSC response to acute kidney injury demonstrated that MDSC reduced the injury, and the effect was potentiated by MDSC induction and enhancement of the immunosuppressive activity promoted by mTOR (22). More recently, a previously unrecognized mechanistic pathway associated with organ rejection identifies the expression of mTOR by graft infiltrating macrophages at the center of epigenetic and metabolic changes that correlate with graft loss (23). This novel functional mechanism involves non-permanent reprogramming of macrophages and has been termed “trained immunity” (24). Therefore, it seems that, while mTOR inhibition may prevent trained immunity and inflammatory pathways in myeloid cells (25, 26), it may also interfere with tolerogenic programming and the ability of myeloid cells to expand Treg and suppress T-cell-mediated immune responses. This dual effect of mTOR inhibition (immunogenic vs. tolerogenic) and the resulting dominant pathway *in vivo* is likely to determine the outcome of the transplanted organ. Taken together, the effects of distinct immunosuppressive drugs on MDSC development and function need to be better characterized in KTR.

Understanding the effect of immunosuppressive drugs on MDSC in clinical transplantation is important to develop strategies to promote tolerance. While there are many unanswered questions regarding the development and function of MDSC human transplantation, we conclude that MDSCs are increased in KTR early after transplantation and that Mo-MDSC subsets from KTR are able to suppress immune responses *in vitro*. How immunosuppressive therapy may enhance or impair MDSC numbers and function is not clear, and additional prospective studies in KTR are required to establish if the long-term transplant tolerance by immune modulation is dependent on MDSC.

## Data Availability Statement

The datasets generated for this study are available on request to the corresponding author.

## Ethics Statement

The studies involving human participants were reviewed and approved by the Hospital Universitario Marqués de Valdecilla Ethics Committee (CEIC). The patients/participants provided their written informed consent to participate in this study.

## Author Contributions

MI-E: data acquisition, analysis, interpretation, investigation, methodology, writing, and original draft. DS-A and PR: conceptualization, formal analysis, supervision, writing, and review. DM-F: data acquisition, analysis, interpretation, investigation and methodology. SG-F, CP, and PL-P: investigation, and methodology. RV, JR, and ER: patient recruitment and clinical data analysis. JH, JO, and ML-H: conceptualization, project administration, funding acquisition, formal analysis, writing, reviewing, and editing.

## Conflict of Interest

The authors declare that the research was conducted in the absence of any commercial or financial relationships that could be construed as a potential conflict of interest.

## Supplementary Material

FIGURE S1 Comparison of MDSC subsets: Mo-MDSC, PMN-MDSC, and e-MDSC at day 0 and 180 days after transplant **(A)** and at day 0, day 180, and 360 after transplant **(B).** Levels of Mo-MDSC 180 days after transplant were significantly increased compared to day 0. The central number is the difference (in percent) between the means of the two time points **(A)** and the three time points **(B)**. Differences between time points were calculated using the following formula: (mean posTx-mean preTx)/mean preTx.

FIGURE S2 MDSC absolute numbers in KTR. **(A)** Frequencies of total myeloid-derived suppressor cells (MDSCs) in peripheral blood mononuclear cells (PBMCs); **(B)** monocytic-MDSCs (Mo-MDSCs); **(C)** early stage-MDSC (eMDSCs), and **(D)** polymorphonuclear MDSCs (PMN-MDSCs) are shown. Differences between groups were assessed by Kruskal-Wallis and Mann-Whitney *U* test. (^∗^*p* < 0.05).

## Abbreviations

7AAD: 7-amino-actinomycin D
CNI: calcineurin inhibitors
e-MDSC: early-stage MDSCs
HC: healthy controls
KTRs: kidney transplant recipients
MDSCs: myeloid-derived suppressor cells
Mo-MDSCs: monocytic MDSCs
mTOR: mammalian target of rapamycin
PBMC: peripheral blood mononuclear cells
PBS: phosphate-buffered saline
PMN-MDSCs: polymorphonuclear MDSCs.
