# Supplementary material for: Myeloid-Derived Suppressor Cells in Kidney Transplant Recipients and the Effect of Maintenance Immunotherapy
Source: Front Immunol. 2020 Apr 30;11:643. doi: 10.3389/fimmu.2020.00643 (PMC7203496; doi:10.3389/fimmu.2020.00643)
Supplement: FIGURE S1 — Comparison of MDSC subsets: Mo-MDSC, PMN-MDSC, and e-MDSC at day 0 and 180 days after transplant (A) and at day 0, day 180, and 360 after transplant (B). Levels of Mo-MDSC 180 days after transplant were significantly increased compared to day 0. The central number is the difference (in percent) between the means of the two time points (A) and the three time points (B). Differences between time points were calculated using the following formula: (mean posTx-mean preTx)/mean preTx. [file Presentation_1.pptx]

## Slide 1
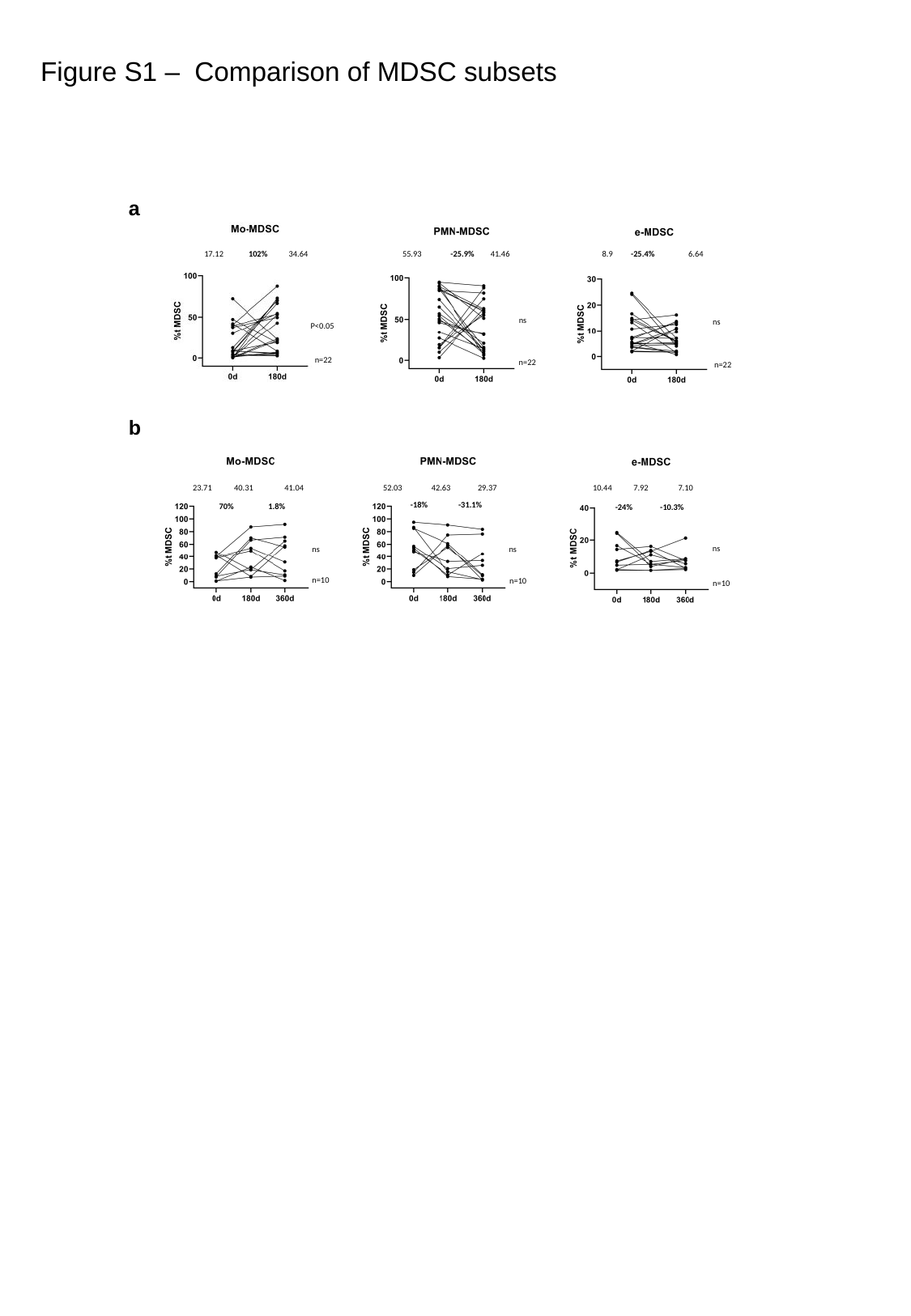

Figure S1 – Comparison of MDSC subsets
 a
17.12
102%
34.64
P<0.05
n=22
-25.9%
-25.4%
41.46
55.93
8.9
6.64
n=22
n=22
ns
ns
 b
23.71
40.31
41.04
52.03
42.63
29.37
10.44
7.92
7.10
-18%
-31.1%
1.8%
70%
-10.3%
-24%
ns
ns
ns
n=10
n=10
n=10

## Slide 2
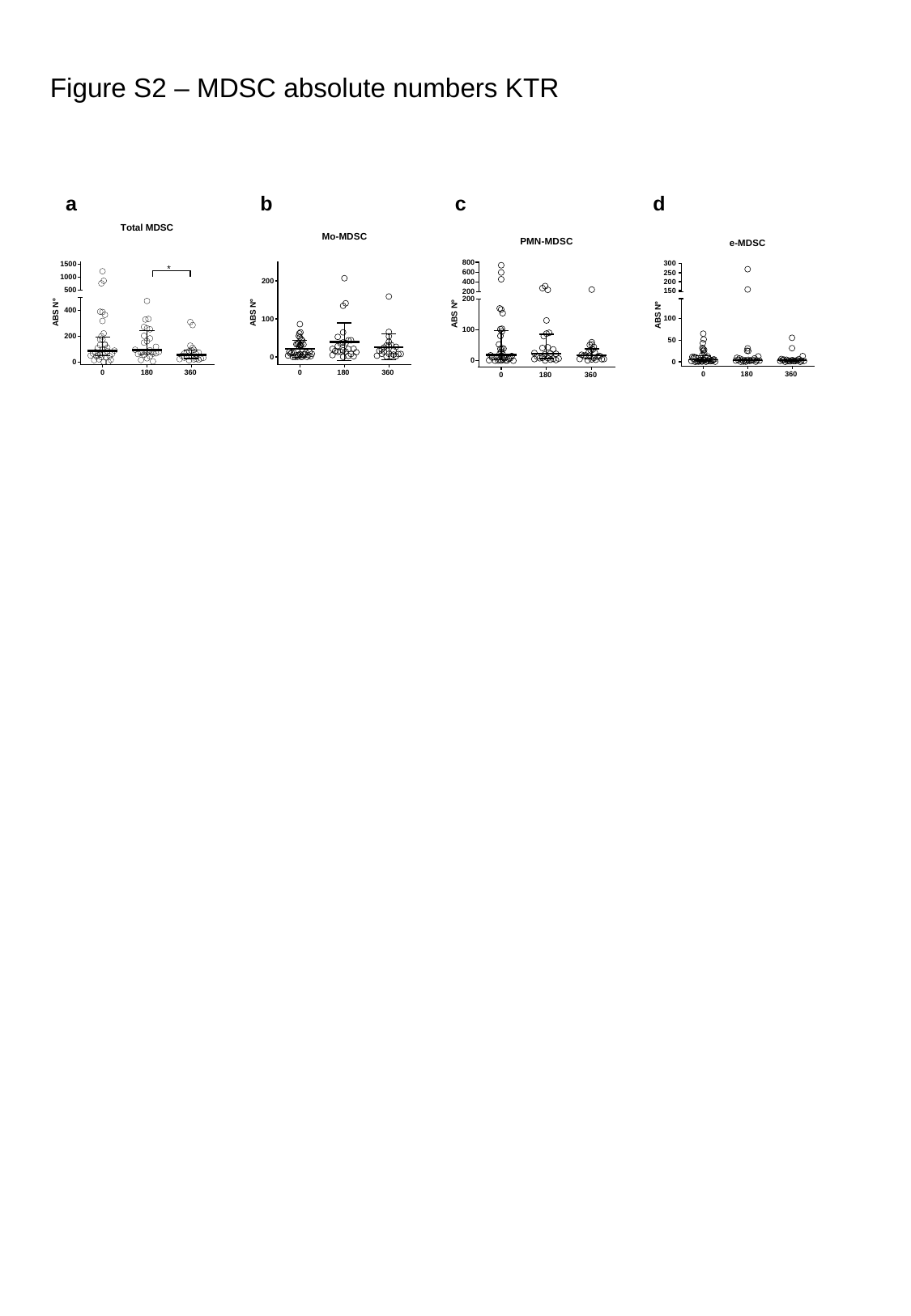

Figure S2 – MDSC absolute numbers KTR
 d
a
c
 b
